# Bacterial, viral, and fungal infection‐related risk of Parkinson's disease: Meta‐analysis of cohort and case–control studies

**DOI:** 10.1002/brb3.1549

**Published:** 2020-02-04

**Authors:** Hui Wang, Xi Liu, Changhong Tan, Wen Zhou, Jin Jiang, Wuxue Peng, Xuan Zhou, Lijuan Mo, Lifen Chen

**Affiliations:** ^1^ Department of Neurology The Second Affiliated Hospital of Chongqing Medical University Chongqing China

**Keywords:** *Helicobacter pylori*, hepatitis C virus, infection, Malassezia, meta‐analysis, Parkinson disease, pneumoniae, risk

## Abstract

**Aims:**

Recent studies showed that patients with various bacterial, viral, and fungal infections might be at increased risk of Parkinson's disease (PD). However, the risk of PD in patients with each specific infection varied. This meta‐analysis estimated the association between various infections and PD risk.

**Methods:**

Literature published from January 1965 to October 2019 in PubMed and EMBASE databases was searched. Data were extracted and pooled using random/fixed effects model. Sensitivity analysis and meta‐regression were also performed to analyze the source of heterogeneity. Publication bias was estimated by the trim and fill.

**Results:**

Twenty‐three out of 6,609 studies were included. *Helicobacter pylori* (HP; pooled OR = 1.653, 1.426–1.915, *p* < .001), hepatitis C virus (HCV; pooled OR = 1.195, 1.012–1.410, *p* = .035), Malassezia (pooled OR = 1.694, 1.367–2.100, *p* < .001), and pneumoniae (pooled OR = 1.595, 1.020–2.493, *p* = .041) infection were associated with increased PD risk. Influenza virus, herpes virus, hepatitis B virus, scarlet fever, mumps virus, chicken pox, pertussis, German measles, and measles were not associated with PD risk. After antiviral treatment against HCV reduced the risk of PD in patients with HCV infection (OR = 0.672, 0.571–0.791, *p* < .001). Significant heterogeneity exists among the included studies.

**Conclusion:**

Patients with infection of HP, HCV, Malassezia, pneumoniae might be an increased risk of PD. Antiviral treatment of HCV could reduce the risk of PD.

## Graphical abstract

Bacterial, viral, or overall infection was associated with increased Parkinson's disease risk. *Helicobacter pylori*, hepatitis C virus, Malassezia, and pneumoniae infection were positively associated with the risk of Parkinson's disease. However, infection of herpes virus, hepatitis B virus, pertussis, scarlet fever, mumps, chicken pox, influenza virus, measles, and German measles was not.

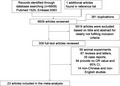

## 1 INTRODUCTION

Parkinson's disease (PD) is a common neurodegenerative disease in the elderly, which is clinically characterized by resting tremor, bradykinesia, rigidity, and postural instability and difficulty in walking (Gerfen, 2000). Approximately 7.5 million people worldwide are affected by Parkinson's disease and the prevalence increases rapidly with age. It has been anticipated that there will be nine million PD patients by 2030 (Pringsheim, Jette, Frolkis, & Steeves 2014; Ross & Abbott, 2014). The main pathological change of PD is degeneration and death of dopaminergic neurons in the substantia nigra due to unclear etiology and pathogenesis (Li et al., 2019; Salamon, Zádori, Szpisjak, Klivényi, & Vécsei, 2019). Similar to other neurodegenerative diseases, multiple factors, including gene, neuroinflammation, trauma, drugs, and toxicity, appear to play important roles in the development of PD (Dick et al., 2007; Mcgeer, Yasojima, & Mcgeer, 2003; Park et al., 2019; Xu, Chen, Xu, Zhang, & Li, 2018; Xu et al., 2014). Recently, infection is increasingly recognized as a risk factor for PD (Liu, Gao, & Hong, 2003; Mattson, 2004) because it may trigger chronic inflammation of the microglia (Alam et al., 2016) and, thus, may promote the onset of PD.

Infection of various pathogenic microorganisms has been thought associated with increased risk of PD, including *Helicobacter pylori* (HP; Shen, Yang, Wu, Zhang, & Jiang, 2017), hepatitis C virus (HCV; Wijarnpreecha, Chesdachai, Jaruvongvanich, & Ungprasert, 2018), Malassezia (Laurence, Benito‐León, & Calon, 2019), *Chlamydophila pneumoniae* (*C. pneumonia*; Bu et al., 2015), hepatitis B virus (HBV; Wijarnpreecha et al., 2018), influenza virus (Vlajinac et al., 2013), measles (Vlajinac et al., 2013), varicella–zoster virus (VZV; Hemling et al., 2003), mumps (Vlajinac et al., 2013), German measles (Vlajinac et al., 2013), pertussis (Vlajinac et al., 2013), scarlet fever (Vlajinac et al., 2013), rheumatic fever (Vlajinac et al., 2013), diphtheria (Vlajinac et al., 2013), cytomegalovirus (CMV; Bu et al., 2015), Epstein–Barr virus (EBV; Bu et al., 2015), herpes virus (HSV; Hemling et al., 2003), and *Borrelia burgdorferi* (*B. burgdorferi*; Bu et al., 2015). However, there are also studies indicated that infection of these pathogenic microorganisms may decrease or does not affect the risk of PD (Harris, Tsui, Marion, Shen, & Teschke, 2012). Considering the high prevalence of these infections, such infection‐related increase of risk of PD may affect a quite large population. For example, HCV was reported to affect 0.3% of population in Spain (Crespo et al., 2019), HP colonizes the gastric mucosa of more than half of the global human population (Suwarnalata et al., 2016), Malassezia was identified on more than 80% lesional skin (Arsic Arsenijevic et al., 2014), HBV affects 2 billion people worldwide and over 360 million chronic carriers (Yankam, Anye, Nkfusai, Shirinde, & Cumber, 2019). Therefore, clarification of the effect of such infection on risk of PD may assist the prevention and prediction of PD and bring benefits to a large population. Further study on the mechanism by which these infections increase risk of PD may also improve our understanding of PD pathophysiology.

Notably, although there is no effective prevention strategy for PD, antiviral therapy against HCV has been reported to reduce the risk of PD in patients with HCV infection (Lin et al., 2019; Su et al., 2019), indicating that treatment against pathogenic microorganisms may be a potentially effective method to prevent PD. However, whether treatment against pathogenic microorganisms other than HCV reduces the risk of PD has not been researched.

This meta‐analysis analyzed the relationship between infection of 13 pathogenic microorganisms and risk of PD, including herpes virus, HBV, pertussis, scarlet fever, influenza, mumps, chicken pox, measles, Malassezia, HCV, HP, pneumonia and German measles. We also analyzed the preventive effect of antiviral therapy against HCV on risk of PD in patients with HCV infection.

## 2 METHODS

### 2.1 Search strategy

Literature reporting potential relevance between infection and risk of PD published in PubMed in English from January 1965 to October 2019 was searched. The search strategy was as follows: (Parkinson Disease[MeSH Term]) AND ((((Infection[MeSH Term]) OR Bacteria[MeSH Term]) OR Viruses[MeSH Term]) OR fungi[MeSH Terms]). EMBASE and CNKI database was also searched for articles published using similar searching strategy. If any pathogenic microorganism was identified associated with PD in articles searched using above strategy, a manual search for the related literature between PD and this pathogenic microorganism species was performed. For example, we identified HP and HCV infection were reported associated with PD using above terms; then, we searched the databases manually using the following terms: ((Parkinson's disease) AND Helicobacter pylori), ((Parkinson's disease) AND Hepatitis C virus). Finally, the reference lists of the included articles were also searched manually to identify additional relevant studies not captured by our database search. We did not contact the authors for unpublished data.

### 2.2 Inclusion and exclusion criteria

The inclusion and exclusion criteria were as follows: (a) Original studies determining relationship between infection and risk of PD published in English or Chinese, including case–control studies, and cohort studies were included for further evaluation, experiment, or studies on animals were excluded; (b) odds radios (ORs; unadjusted), relative risks (RRs), and hazard ratios (HRs) with 95% confidence intervals (CI) should be provided or could be calculated in included studies.

### 2.3 Quality assessment of included studies

The quality of each included study was assessed separately by two investigators (H.W. and L.M.) using the validated Newcastle–Ottawa quality assessment scale (NOS; Stang, 2010) in the following three aspects: (a) selection of the participants, (b) comparability between the groups, and (c) ascertainment of the exposure of interest for case–control studies or the outcome of interest for cohort studies. Disagreements of assessment between the two investigators were resolved by the third investigator (X.L.).

### 2.4 Data extraction

A standardized data collection form was used to extracted the following data from each study: (a) last name of the first author, (b) publication year, (c) number of people in PD group and control group, (d) mean age and/or age range, (e) sex in PD group and control group study design, (f) Hoehn–Yahr (H‐Y) stage, (g) PD diagnostic criteria, (h) ORs (unadjusted), RRs, HRs with 95% CIs, (i) infected pathogenic microorganisms, (j) methods for detection of infection, (k) samples used to detect infection, and (l) outcome index. If one study reported data of infection of multiple pathogenic microorganisms, data of each pathogenic microorganism were regarded as an independent study.

### 2.5 Statistical analysis

All statistical analyses were performed using Stata 12.0 software (StataCorp LP). *p* < .05 was defined as statistically significant. Pooled OR with 95% CI was calculated using random/fixed effect model to assess the association of bacteria, virus, and fungus infections with the risks of PD. If *I* ^2^ < 50%, the fixed effects model was selected; otherwise, the random effects model was selected. Heterogeneity was determined by the *I* ^2^ statistic (*I* ^2^ > 50% was considered with significant heterogeneity). The contribution of publication year, mean age and/or age range, study design, PD diagnostic criteria, infected pathogenic microorganisms, methods for detection of infection, and samples used to detect infection to heterogeneity were analyzed by sensitivity analysis. If sensitivity analysis failed to identify the source of heterogeneity, and if there are more than three articles on this pathogenic microorganism, meta‐regression was performed to identify the source of heterogeneity. Trim and fill method was used to assess the publication bias.

## 3 RESULTS

### 3.1 Search result and study characteristics

**Figure 1:**
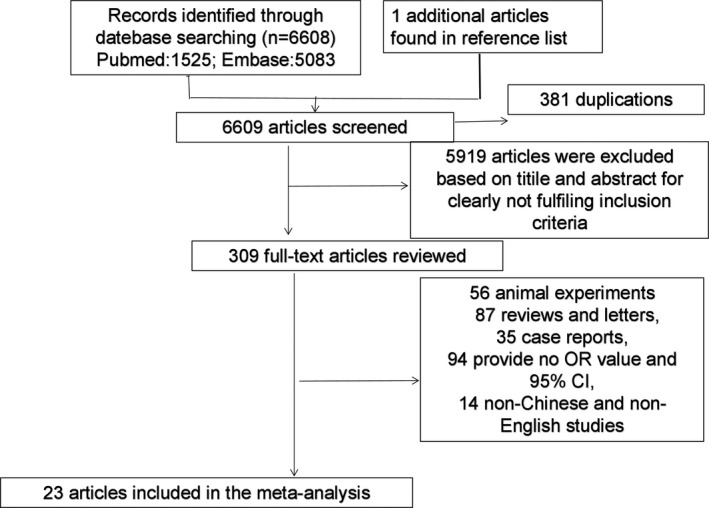
Flowchart of literature screening

A total of 6,609 possibly relevant articles were identified in PubMed and EMBASE database by our search strategy and manual searching; no relevant article published in Chinese was identified in CNKI database. As shown in Figure 1, 23 observational studies (Arsic Arsenijevic et al., 2014; Blaecher et al., 2013; Bu et al., 2015; Charlett et al., 1999, 2009; Dobbs, Charlett, Dobbs, Weller, & Peterson, 2000; Golabi et al., 2017; Goldstein, Fogel‐Grinvald, & Steiner, 2019; Harris et al., 2012; Hemling et al., 2003; Huang et al., 2018; Kim et al., 2016; Nafisah et al., 2013; Nielsen, Qiu, Friis, Wermuth, & Ritz, 2012; Pakpoor et al., 2017; Sasco & Paffenbarger, 1985; Su et al., 2019; Tanner et al., 2012; Toovey, Jick, & Meier, 2011; Tsai et al., 2016; Tsolaki, Kountouras, Topouzis, & Tsolaki, 2015; Vlajinac et al., 2013; Wu et al., 2015) including six retrospective cohort study and 17 case–control studies published from 1965 to 2019 were included. Among these 23 studies, infection of 21 pathogenic microorganisms including eight bacteria, 12 viruses and one fungus were reported (Table 1). Among these reported pathogenic microorganisms, *B. burgdorferi*, tuberculosis, diphtheria, rheumatic fever, cytomegalovirus (CMV), Epstein–Barr virus (EBV), human herpes virus 6 (HHV‐6), varicella–zoster virus (VZV), and red measles were only reported in one study, which was insufficient for meta‐analysis. Therefore, this meta‐analysis pooled the data on infection of herpes virus, HBV, pertussis, scarlet fever, influenza, mumps, chicken pox, measles, Malassezia, HCV, HP, pneumonia and German measles.

**Table 1:** Characteristics of included studies

Study	Study design	Infection	Case (PD/non‐PD)	Control (PD/non‐PD)	OR (95% CI)	H‐Y stage	Detection method	Sample	Diagnostic criteria of PD
Bu 2015	Case–control	HP	60/131	44/141	1.787 (1.081–2.959)	2.24 (0.91)	ELISA	Serum	UK PD Society Brain Bank
Bu 2015	Case–control	*Borrelia burgdorferi*	23/131	11/141	2.466 (1.139–5.339)	2.24 (0.91)	ELISA	Serum	UK PD Society Brain Bank
Bu 2015	Case–control	HSV‐1	115/131	111/141	1.92 (0.981–3.759)	2.24 (0.91)	ELISA	Serum	UK PD Society Brain Bank
Bu 2015	Case–control	EBV	130/131	137/141	3.653 (0.395–33.753)	2.24 (0.91)	ELISA	Serum	UK PD Society Brain Bank
Bu 2015	Case–control	CMV	112/131	113/141	1.405 (0.735–2.685)	2.24 (0.91)	ELISA	Serum	UK PD Society Brain Bank
Bu 2015	case–control	*Chlamydophila pneumoniae*	111/131	102/141	2.057 (1.112–3.804)	2.24 (0.91)	ELISA	Serum	UK PD Society Brain Bank
Hemling 2003	Case–control	HSV‐1	33/40	30/40	0.4 (0.1–1.5)	NA	PCR	Brain tissue	Postmortem examination
Hemling 2003	Case–control	VZV	24/40	29/40	1.6 (0.6–4.2)	NA	PCR	Brain tissue	Postmortem examination
Hemling 2003	Case–control	HHV‐6	10/40	5/40	0.5 (0.1–1.6)	NA	PCR	Brain tissue	Postmortem examination
Lilach 2019	Cohort	HBV	NA	NA	1.08 (1.00–1.16)	NA	Clinical	Database	NA
Lilach 2019	Cohort	HCV	NA	NA	1.18 (1.04–1.35)	NA	Clinical	Database	NA
Arsenijevic 2014	Case–control	Malassezia	NA	NA	1.89 (0.45–8.02)	NA	CFU/tape	NA	Clinical
Vlajinac 2013	Case–control	Tuberculosis	2/110	4/220	1.00 (0.18–5.55)	NA	Questionnaire	NA	Clinical
Vlajinac 2013	Case–control	Measles	35/110	61/220	1.22 (0.74–2.00)	NA	Questionnaire	NA	Clinical
Vlajinac 2013	Case–control	Pertussis	7/110	1/220	14.86 (1.81–121.95)	NA	Questionnaire	NA	Clinical
Vlajinac 2013	Case–control	Influenza	70/110	42/220	7.42 (4.44–12.39)	NA	Questionnaire	NA	Clinical
Vlajinac 2013	Case–control	Mumps	35/110	12/220	8.09 (3.99–16.39)	NA	Questionnaire	NA	Clinical
Vlajinac 2013	Case–control	Chicken pox	35/110	82/220	0.78 (0.48–1.28)	NA	Questionnaire	NA	Clinical
Vlajinac 2013	Case–control	Herpes virus	10/110	2/220	10.89 (2.34–50.76)	NA	Questionnaire	NA	Clinical
Vlajinac 2013	Case–control	Scarlet fever	6/110	2/220	6.29 (1.25–31.65)	NA	Questionnaire	NA	Clinical
Nielsen 2012	Case–control	HP	138/4,484	505/22,416	1.46 (1.210–1.770)	NA	Clinical	NA	Based on PD drug administration
Blaecher 2013	Case–control	HP	17/60	42/256	2.014 (1.050–3.865)	NA	PCR	Serum	NA
Dobbs 2000	Case–control	HP	25/58	43/136	2.04 (1.040–4.220)	NA	ELISA	Serum	NA
Nafisah 2013	Case–control	HP	14/29	5/23	3.36 (0.982–11.492)	NA	Clinical	NA	NA
Tsolaki 2015	Case–control	HP	6/9	14/31	2.492 (0.512–11.511)	NA	Clinical	NA	NA
Kim 2016	Case–control	HBV	47/1,558	59/1,558	0.79 (0.54–1.17)	NA	ELISA	NA	Clinical
Kim 2016	Case–control	HCV	27/1,558	14/1,558	1.95 (1.02–3.72)	NA	ELISA	NA	Clinical
SASCO 1985	Case–control	Diphtheria	9/137	36/548	1.0 (0.48–2.2)	NA	Questionnaire	NA	Questionnaires or death certificates
SASCO 1985	Case–control	Pertussis	77/137	329/548	0.88 (0.60–1.3)	NA	Questionnaire	NA	Questionnaires or death certificates
SASCO 1985	Case–control	Measles	112/137	494/548	0.53 (0.31–0.93)	NA	Questionnaire	NA	Questionnaires or death certificates
SASCO 1985	Case–control	Mumps	62/137	271/548	0.88 (0.60–1.3)	NA	Questionnaire	NA	Questionnaires or death certificates
SASCO 1985	Case–control	Influenza	32/137	118/548	1.1 (0.72–1.8)	NA	Questionnaire	NA	Questionnaires or death certificates
SASCO 1985	Case–control	Rheumatic fever	4/137	9/548	1.8 (0.56–6.0)	NA	Questionnaire	NA	Questionnaires or death certificates
SASCO 1985	Case–control	Chicken pox	112/137	347/548	0.76 (0.52–1.1)	NA	Questionnaire	NA	Questionnaires or death certificates
SASCO 1985	Case–control	German measle	4/137	11/548	1.8 (0.38–8.8)	NA	Questionnaire	NA	Questionnaires or death certificates
CharlettA 1999	Case–control	HP	23/33	31/78	3.04 (1.220–7.630)	NA	ELISA	Serum	Clinical
CharlettA 2009	Cross‐sectional	HP	57/120	77/196	1.398 (0.884–2.212)	NA	ELISA	Serum	Clinical
Huang 2017	Cohort	HP	64/9105	25/9,105	2.65 (1.67–4.20)	NA	Biopsy	Esogastritis	Clinical
Golabi 2017	Cohort	HBV	23/1,300	16,004/1,228,849	1.365 (0.903–2.062)	NA	ELISA	Serum	Clinical
Golabi 2017	Cohort	HCV	49/6,040	16,004/1,228,849	0.62 (0.468–0.821)	NA	ELISA	Serum	Clinical
Pakpoor 2017	Cohort	HBV	25/44	NA	1.76 (1.28–2.37)	NA	NA	NA	Clinical
Pakpoor 2017	Cohort	HCV	48/73	NA	1.51 (1.18–1.90)	NA	NA	NA	Clinical
Wu 2015	Case–control	HBV	2,664/62,276	843/24,762	0.62 (0.48–0.81)	NA	PCR	Serum	Clinical
Wu 2015	Case–control	HCV	7,076/62,276	3,156/24,762	1.39 (1.07–1.81)	NA	PCR	Serum	Clinical
Tsai 2015	Cohort	HBV	121/35,498	NA	0.66 (0.55–0.80)	NA	NA	NA	Clinical
Tsai 2015	Cohort	HCV	120/10,286	NA	2.5 (2.07–3.02)	NA	NA	NA	Clinical
Su 2019	Cohort	HCV	797/248,647	1,100/385,791	1.176 (1.070–1.292)	NA	ELISA	Serum	Clinical
Harris 2012	Case–control	Influenza	43/403	26/405	2.01 (1.16–3.48)	NA	Questionnaire	NA	Clinical
Harris 2012	Case–control	German measles	43/403	49/405	1.27 (0.77–2.08)	NA	Questionnaire	NA	Clinical
Harris 2012	Case–control	Mumps	208/403	232/405	0.76 (0.56–1.02)	NA	Questionnaire	NA	Clinical
Harris 2012	Case–control	Herpes virus	43/403	49/405	0.96 (0.60–1.53)	NA	Questionnaire	NA	Clinical
Harris 2012	Case–control	Red measles	242/403	291/405	0.65 (0.48–0.90)	NA	Questionnaire	NA	Clinical
Harris 2012	Case–control	Chicken pox	252/403	296/405	0.75 (0.54–1.03)	NA	Questionnaire	NA	Clinical
Toovey 2011	Case–control	Influenza	28/3,753	126/15,891	0.91 (0.60–1.38)	NA	Questionnaire	NA	Clinical
Tanner 2016	Case–control	Malassezia	115/2,651	333/13,255	1.69 (1.36–2.1)	NA	Clinical	NA	Clinical

Abbreviations: CFU/tape, colony forming units per tape; CMV, cytomegalovirus; EBV, Epstein–Barr virus; ELISA, enzyme‐linked immunosorbent assay; HBV, hepatitis B virus; HCV, hepatitis C virus; HHV‐6, human herpesvirus 6; HP, *Helicobacter pylori*; HSV‐1, herpes simplex virus type‐1; NA, not applicable; NOS, Newcastle–Ottawa Scale; OR, odds ratio; PCR, polymerase chain reaction; PD, Parkinson's disease; VZV, varicella–zoster virus.

### 3.2 Literature quality assessment using NOS

Each included study was assessed using NOS; all studies have a NOS score more than 7, indicating a favorable quality of included studies. Detailed NOS score of each included study was listed in Table 2.

**Table 2:** Quality assessment of included studies based on Newcastle–Ottawa scale

Study	Selection	Comparability	Outcome	NOS
Bu 2015	3	2	3	8
Hemling 2003	3	2	3	8
Lilach 2019	4	2	3	9
Arsenijevic 2014	3	2	3	8
Vlajinac 2013	3	2	3	8
Nielsen 2012	4	1	3	8
Blaecher 2013	3	2	3	8
Dobbs 2000	3	2	3	8
Nafisah 2013	4	2	3	9
Tsolaki 2015	3	2	3	8
Kim 2016	4	2	3	8
SASCO 1985	3	2	3	8
CharlettA 1999	3	2	3	8
CharlettA 2009	4	2	3	9
Huang 2017	4	2	3	9
Golabi 2017	4	2	2	8
Pakpoor 2017	4	1	2	7
Wu 2015	4	2	3	9
Tsai 2015	3	2	3	8
Su 2019	4	2	3	9
Harris 2012	4	2	2	8
Toovey 2011	3	2	3	8
Tanner 2016	4	2	2	8

HP, HCV, Malassezia and pneumoniae was positively associated with risk of PD. Antiviral treatment of HCV reduced the risk of PD.

**Figure 2:**
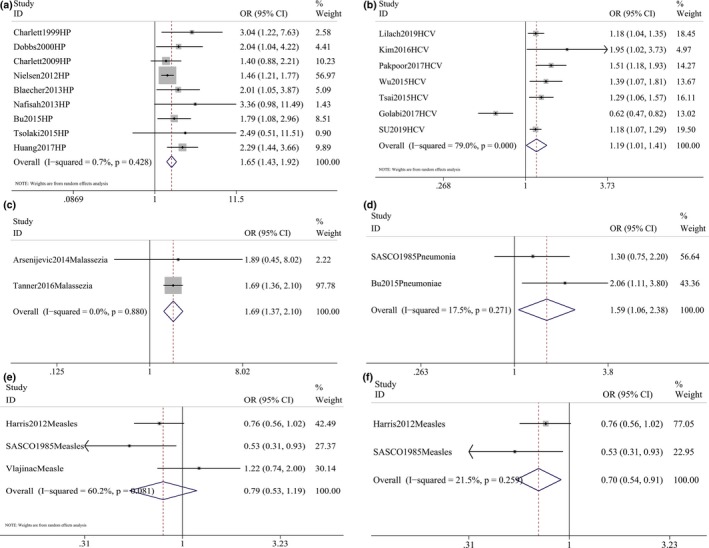
Forest plot for the pooled odds ratios (ORs) showed positive association between *Helicobacter pylori* (HP; a), hepatitis C virus (HCV; b), Malassezia (c), pneumoniae (d), and measles (f) the risk of Parkinson's disease (PD). Pooled OR of measles showed significant association with risk of PD after excluding one study that was responsible for heterogeneity (e)

**Figure 3:**
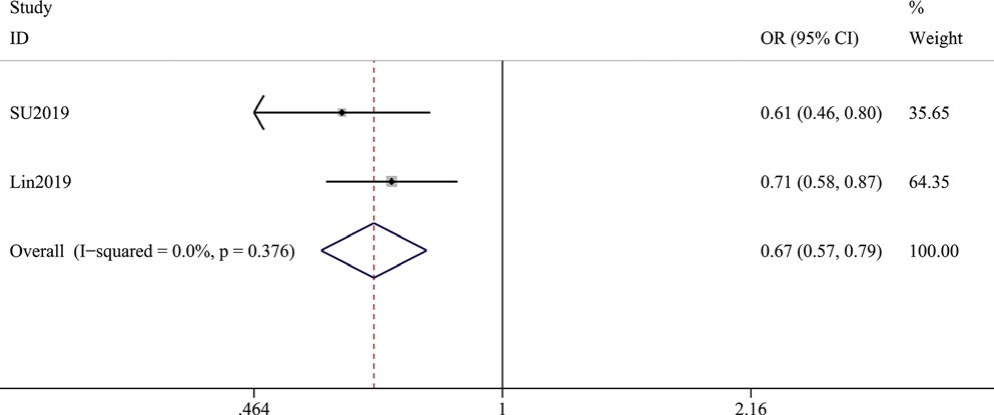
Forest plot for the pooled odds ratios (ORs) of antiviral treatment against hepatitis C virus (HCV) showed antiviral treatment against HCV significant reduced the risk of Parkinson's disease (PD)

We analyzed the relationship between infection of each specific pathogenic microorganisms and the risk of PD. The pooled results showed that HP (pooled OR = 1.653, 1.426–1.915, *p* < .001), HCV (pooled OR = 1.195, 1.012–1.410, *p* = .035), Malassezia (pooled OR = 1.694, 1.367–2.100, *p* < .001), and pneumoniae (pooled OR = 1.595, 1.020–2.493, *p* < .001) were positively associated with the risk of PD, and only low heterogeneity was identified (*I* ^2^ = 0.7%, *p* = .428; *I* ^2^ = 79%, *p* < .001; *I* ^2^ = 0.0%, *p* = .880; *I* ^2^ = 17.5%, *p* = .271, respectively; Figure 2). A positive association between antiviral treatment against HCV and risk of PD was also identified (pooled OR = 0.672, 0.571–0.791, *p* < .001) with very low heterogeneity (*I* ^2^ = 0.0%, *p* = .376; Figure 3).

### 3.3 Infection of influenza virus, herpes virus, HBV, scarlet fever, mumps, chicken pox, pertussis, German measles, and measles virus were not associated with risk of PD

**Figure 4:**
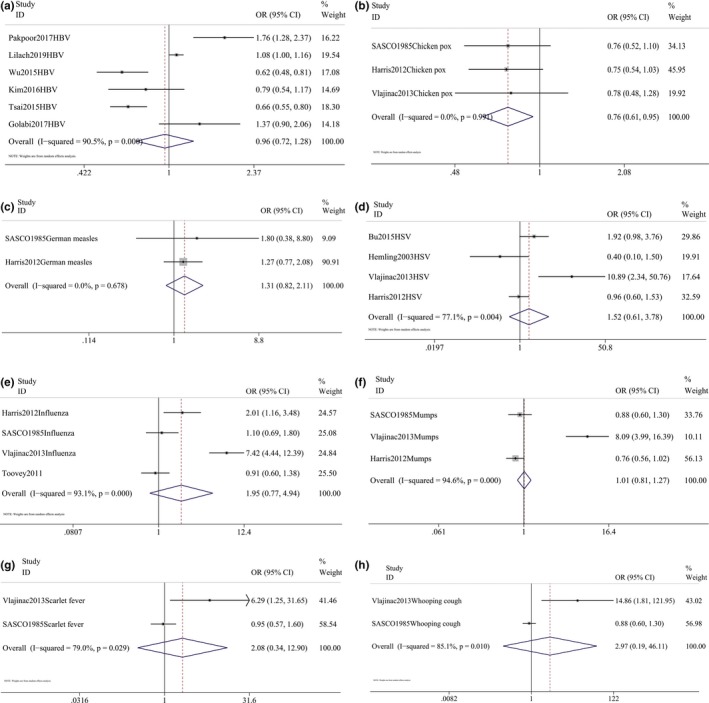
Forest plot for the pooled odds ratios (ORs) showed that hepatitis B virus (HBV; a), chicken pox (b), German measles (c), herpes virus (HSV; d), influenza virus (e), mumps (f),scarlet fever (g), and whooping cough (pertussis; h) infection had no association with the risk of Parkinson's disease (PD)

The meta‐analysis found that influenza virus (pooled OR = 1.953, 0.772–4.939, *p* = .157, *I* ^2^ = 93.1%, *p* < .001), herpes virus (pooled OR = 1.522, 0.613–3.779, *p* = .365, *I* ^2^ = 77.1%, *p* = .004), HBV (pooled OR = 0.959, 0.716–1.285, *p* = .781, *I* ^2^ = 90.5%, *p* < .001), scarlet fever (pooled OR = 2.080, 0.335–12.905, *p* = .432, *I* ^2^ = 79%, *p* = .029), mumps virus (pooled OR = 1.662, 0.572–4.826, *I* ^2^ = 94.6%, *p* < .001), chicken pox (pooled OR = 0.759, 0.610–0.945, *p* = .014, *I* ^2^ = 0.0%, *p* = .991), pertussis (pooled OR = 2.969, 0.191–46.111, *p* = .437, *I* ^2^ = 85.1%, *p* = .010), German measles (pooled OR = 1.311, 0.816–2.105, *p* = .263, *I* ^2^ = 0.0%, *p* = .678), and measles (pooled OR = 0.794, 0.530–1.189, *p* = .263, *I* ^2^ = 60.2%, *p* = .081) were not significantly associated with risk of PD (Figure 4).

### 3.4 Sensitivity analysis and meta‐regression

No significant heterogeneity exists among studies on HP, Malassezia, pneumoniae, chicken pox, and German measles.

**Figure 5:**
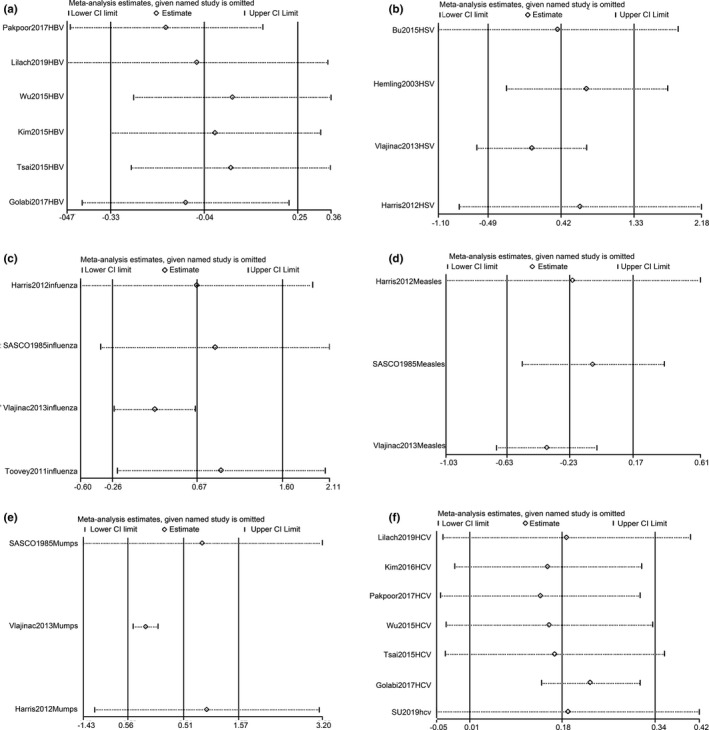
Sensitivity analysis for studies on hepatitis B virus (HBV; a), herpes virus (HSV; b), influenza virus (c), measles (d), mumps (e), and hepatitis C virus (HCV; f) for the risk of Parkinson's disease (PD)

Due to the high heterogeneity when pooled ORs of HBV, HCV, influenza virus, mumps, herpes virus, and measles, we performed sensitivity analysis to identify the source of heterogeneity. Sensitivity analysis found that excluding the study by Vlajinac et al. (2013) from the pooled analysis could significant reduce the heterogeneity of studies on influenza virus, mumps, and measles. This may be because that Vlajinac's study (Vlajinac et al., 2013) used a structured questionnaire to gained personal and family histories, which may affect the accuracy of infection history. After excluding Vlajinac's study (Vlajinac et al., 2013), the pooled OR of influenza virus, herpes virus, and mumps was not significantly changed (influenza virus: pooled OR = 1.227, 0.786–1.917, *p* = .175, *I* ^2^ = 61.5%; herpes virus: pooled OR = 1.062, 0.534–2.111, *p* = .864, *I* ^2^ = 60.9%; mumps: pooled OR = 0.803, 0.634–1.018, *p* = .070, *I* ^2^ = 0.0%), while the poole OR of measles virus was significantly changed (pooled OR = 0.685, 0.498–0.943, *p* = .020, *I* ^2^ = 21.5%), indicating a negative association between measles infection and risk of PD. Significant heterogeneity was identified among studies on HCV (*I* ^2^ = 79%, *p* < .001), and sensitivity analysis found that Golabi's study (Golabi et al., 2017) accounted for the source of heterogeneity, excluding Golabi's study (Golabi et al., 2017) did not change the outcome of meta‐analysis (*I* ^2^ = 27.8%, *p* = .227). Sensitivity analysis failed to identify the source of heterogeneity among studies on HBV (Figure 5).

**Figure 6:**
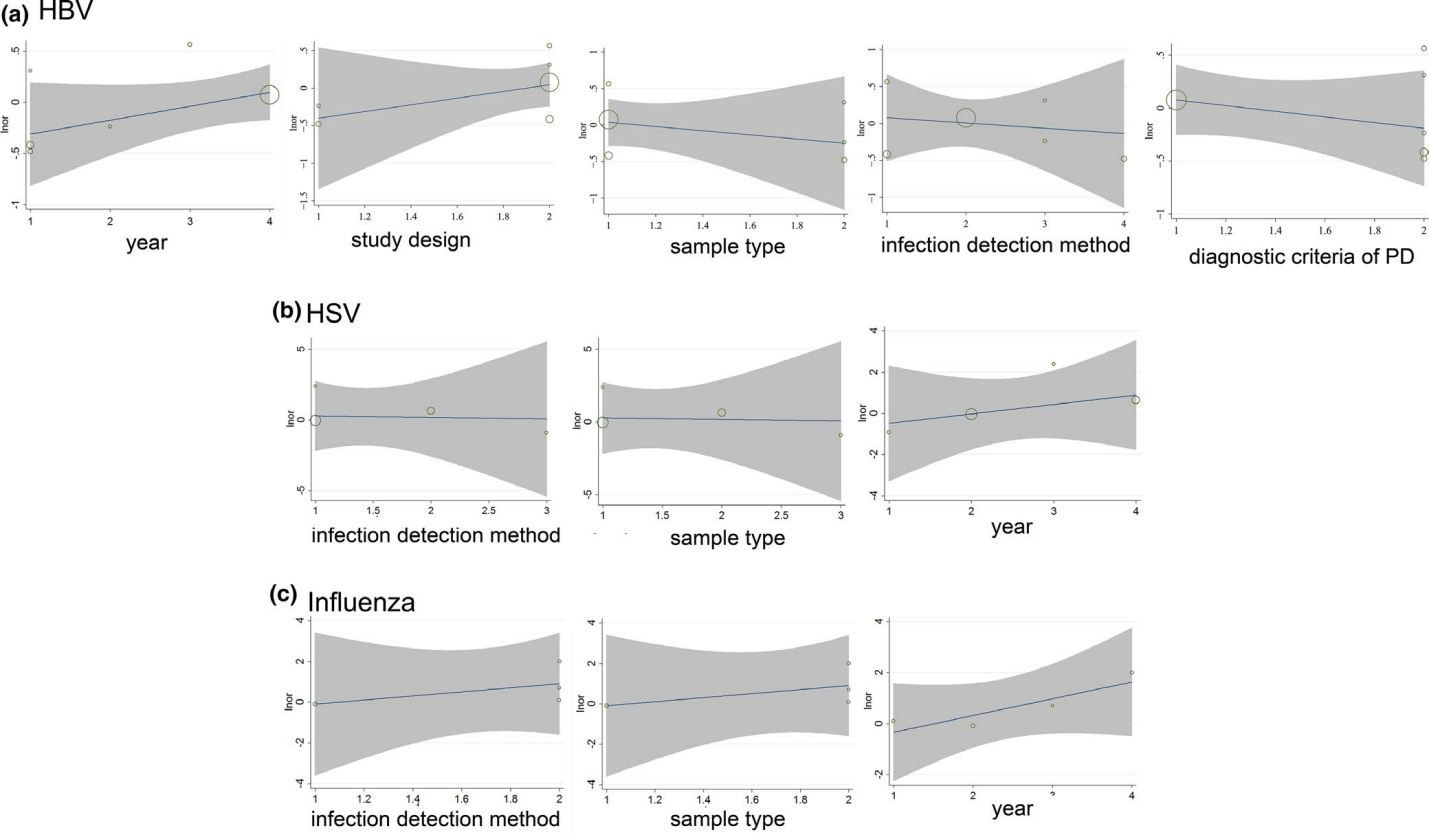
Meta‐regression analysis for studies on hepatitis B virus (HBV; a), herpes virus (HSV; b), and influenza virus (c) based on year of publication, study design, sample type, infection detection methods, and diagnostic criteria of Parkinson's disease (PD)

For those pathogenic microorganisms which sensitivity analysis failed to identify the source of their heterogeneity, including HBV, HSV, and influenza virus, meta‐regression analysis was performed based on factors including the publication year, study design, diagnostic criteria of PD and infection detection method. However, None of the above factors was identified as the source of heterogeneity (Figure 6).

For scarlet fever and pertussis, only two studies were included, which is insufficient for sensitivity analysis or meta‐regression.

### 3.5 Analysis of publication bias

**Figure 7:**
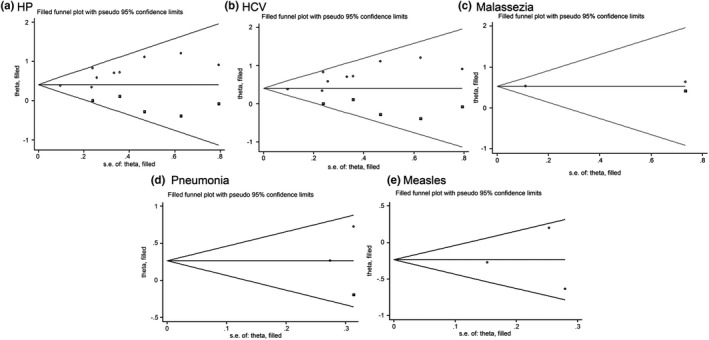
Publication bias analysis of studies on *Helicobacter pylori* (HP; a), hepatitis C virus (HCV; b), Malassezia (c), pneumoniae (d), and measles (e) infection estimated by Trill and Filled methods showed significant publication bias

The results of trim and fill method were shown in Figure 4. Funnel plots of HP (*z* = −0.059, *p* < .001), HCV (*z* = 6.122, *p* < .001), HSV (*z* = 0.906, *p* = .365), chicken pox (*z* = −2.466, *p* = .014), German measles (*z* = 1.120, *p* = .263), Malassezia (*z* = 4.811, *p* < .001), and pneumoniae (*z* = 2.233, *p* = .026) infection showed significant asymmetry, suggesting significant publication bias exists. Funnel plots of HBV (*z* = −0.278, *p* = .781), influenza virus (*z* = 1.414, *p* = .157), mumps (*z* = 0.933, *p* = .351), measles (*z* = −1.119, *p* = .0263), scarlet fever (*z* = 0.786, *p* = .432), and pertussis (*z* = 0.778, *p* = .437) infection did not show significant asymmetry, suggesting that no significant publication bias presented (Figure 7).

## 4 DISCUSSION

This meta‐analysis analyzed infection‐related risk of PD, including HP, HCV, HBV, Malassezia, pneumoniae, chicken pox, German measles, influenza virus, herpes virus, mumps, measles, pertussis, and scarlet fever. The results of this meta‐analysis showed HP, HCV, Malassezia, and pneumoniae may increase the risk of PD, while influenza virus, herpes virus, HBV, scarlet fever, mumps, chicken pox, pertussis, German measles, and measles virus were not significantly associated to risk of PD. Notably, after sensitivity analysis, measles showed a negative association with risk of PD after excluding Vlajinac's study (Vlajinac et al., 2013). Additionally, this meta‐analysis, antiviral therapy against HCV could reduce the risk of PD. The NOS score of each included study was more than 7, indicating favorable quality of included studies.

In this meta‐analysis, we found that infection of HP, HCV, Malassezia, and pneumoniae was positively associated with the risk of PD. HP infection has been found to increase the synthesis of MPTP or MPTP‐like substance (Altschuler, 1996) and cause chronic inflammation in central nervous system which damage dopaminergic neurons (Hirai et al., 1995) via activating microglia (Streit, Mrak, & Griffin, 2004), releasing neurotoxic substances (Villarán et al., 2010), or inducing autoimmune responses (Dobbs et al., 1999). HP infection may also affect symptoms of PD via decreasing absorption of levodopa and was related to poorer motor function in PD patients (Shen et al., 2017; Suwarnalata et al., 2016). Therefore, HP infection may be a potential causal factor of PD onset. In clinic, it may be reasonable to consider screening and eradicating HP in patients with family history of PD or at high risk of PD, especially considering the high prevalence of HP infection. In patients with PD, eradication of HP may alleviate motor symptoms or strengthen effect of levodopa, but whether eradicating HP affect the natural process or progression of PD remains to be further researched.

Hepatitis C virus has been reported to increase risk of PD (Kim et al., 2016); a previous meta‐analysis also reported increased PD incidence in patients with HCV infection (Wijarnpreecha et al., 2018). HCV has been reported to cause PD by inducing inflammatory cytokine release and damaging dopaminergic neurons (Alam et al., 2016; Mattson, 2004). It has been reported that the essential HCV receptors such as CD81, claudin‐1, occludin, LDLR, and scavenger receptor‐B1 are expressed on brain microvascular endothelial cells, a major component of the blood–brain barrier, suggesting that HCV may infect the central nervous system through these receptors (Alam et al., 2016; Fletcher et al., 2012). HCV‐induced inflammatory cytokines release may also contribute to the pathogenesis of PD. In animal models, HCV induced 60% of dopaminergic neuron death in rat midbrain (Alam et al., 2016). In patients, the toxic effect of HCV on dopaminergic neurons was found similar to 1‐methyl‐4‐phenylpyridinium (MPP+), and increased the risk of PD (Alam et al., 2016). Our meta‐analysis showed that the risk of PD in HCV patients received effective antiviral treatment against HCV is lower than those who did not, supporting that HCV may be a risk factor for PD (Lin et al., 2019; Su et al., 2019) and that antiviral treatment against HCV could reduce the risk of PD (Lin et al., 2019). Therefore, effective and more active antiviral treatment should be considered in HCV patients; the association between load of HCV and risk of PD still needs to be further researched. Notably, reports showed that receiving interferon‐based antiviral therapy for HCV increased the risk of PD, this may be due to increased drug‐induced parkinsonism in patients receiving interferon therapy (Lin et al., 2019).

In our meta‐analysis, Malassezia infection was related to increased risk of PD (Laurence et al., 2019). Recent studies have shown that PD is associated with seborrheic dermatitis (Laurence et al., 2019), which is known to affect approximately 52%–59% PD patients, and is caused by the excessive proliferation of the lipophilic fungus Malassezia (Laurence et al., 2019). Interestingly, Malassezia has been identified in central nervous system, indicating a possibility that Malassezia may directly contribute to PD (Laurence et al., 2019). However, whether Malassezia increases risk of PD is uncertain, because all the studies on the association between Malassezia and risk of PD are case–control studies. And it has been reported that stiffness of facial expressive muscles in PD patients may contribute to increased sebum accumulation (Laurence et al., 2019) and thus facilitate the infection of Malassezia. The increase of α‐melanocyte‐stimulating hormone and decrease of melanocyte‐stimulating hormone‐inhibiting factor in PD patients may also increase sebum production and Malassezia infection (Laurence et al., 2019). Therefore, Malassezia infection may be just a comorbidity of PD rather than cause of PD. Whether Malassezia infection contributes to increased risk of PD needs to be further researched by cohort studies.

Similar to Malassezia infection, our meta‐analysis and other reports also related Pneumoniae infection to increased risk of PD (Bu et al., 2015). However, these results may be attributed to increased pneumonia incidence in PD patients due to oropharyngeal dysphagia‐induced aspiration or movement disorder‐induced hypostatic pneumonia (Mamolar Andrés et al., 2017; Miyazaki, Arakawa, & Kizu, 2002). Whether infection of pneumoniae could increase the risk of PD still needs to be further investigated.

Additionally, previous studies reported that infection of pertussis, scarlet fever, HBV, herpes virus, influenza virus, mumps, and measles increased the risk of PD (Bu et al., 2015; Harris et al., 2012; Hemling et al., 2003; Sasco & Paffenbarger, 1985; Vlajinac et al., 2013); however, our meta‐analysis did not found significant association between infection of these pathogenic microorganisms and risk of PD possibly due to the limited number of studies. The relation between these pathogenic microorganisms and risk of PD needs to be further studied.

Additionally, infection of EBV (Bu et al., 2015), CMV (Bu et al., 2015), tuberculosis (Vlajinac et al., 2013), HHV‐6 (Hemling et al., 2003), VZV (Hemling et al., 2003), diphtheria (Sasco & Paffenbarger, 1985), rubella (Sasco & Paffenbarger, 1985), and rheumatic fever (Sasco & Paffenbarger, 1985) were also reported associated with risk of PD; however, there were insufficient studies for meta‐analysis. More studies are needed to clarify the role of these pathogenic microorganisms in PD. Notably, the mechanisms by which these pathogenic microorganisms increase the risk of PD onset have never been researched before.

Significant heterogeneity exists in meta‐analyses on influenza virus, herpes virus, HBV, scarlet fever, mumps, chicken pox, pertussis, German measles, and measles virus in this study. Sensitivity analysis showed that heterogeneity when meta‐analyzing influenza virus, herpes virus, measles, and mumps could be completely or partially attributed to Vlajinac's study (Vlajinac et al., 2013). This may be because that Vlajinac's study (Vlajinac et al., 2013) determined infection by questionnaire rather than medical record or laboratory examination, which may induce recall bias and overestimation of viral infection. Interestingly, when we excluded Vlajinac's study (Vlajinac et al., 2013), the meta‐analysis found a significantly decrease risk of PD in patients with measles infection, which was in consistent with previous studies (Harris et al., 2012). It is considered that Measles infection in younger age may induce immune response which was speculated to protect the infected person from other viral infection‐induced substantia nigra damage and thus decreased the risk of PD (Sasco & Paffenbarger, 1985). However, the specific mechanism of this protective effect of measles infection has not been clearly clarified. Meta‐regression failed to identify the source of heterogeneity, indicating that factors other than the collected data may induce heterogeneity in this meta‐analysis. For infection of pertussis and scarlet fever, there were only two studies on each infection, which is insufficient for analysis on heterogeneity. Additionally, although significant heterogeneity exists among studies on HCV infection which could be mainly attributed to Golabi's study (Golabi et al., 2017), we failed to figure out the specific reason of heterogeneity.

Infection of other pathogenic microorganisms was also reported to induce postencephalitic parkinsonism affecting bilateral substantia nigra, including Coxsackie virus, Japanese encephalitis B virus, West Nile virus, Louis encephalitis virus, HIV, Plasmodium falciparum, enteroviruses, and dengue viral infections (Bopeththa & Ralapanawa, 2017; Dourmashkin, Dunn, Castano, & McCall, 2012; He, Yuan, Zhang, & Han, 2015). It is possible that infection of these pathogenic microorganisms may also induce PD by a chronic and gradual process, for example, chronic inflammation in brain (Fletcher et al., 2012). However, none pathogenic microorganism known to induce postencephalitic parkinsonism has been found to positively associated with risk of PD in our meta‐analysis. This suggests that infection of pathogenic microorganisms may have complicated roles in the pathogenesis of PD, and may also affect PD‐related structures other than substantia nigra. More researches are needed to illustrate the role of infection in PD pathogenesis.

Several limitations exist in this study. First, data on infection of many included pathogenic microorganisms could not be pooled due to the limited number of studies. Second, only studies published in English and Chinese were included; this may induce bias. Third, the diagnostic criteria of PD in the included studies differed, which may affect the estimation of risk of PD. Forth, diagnosis of infection in the included studies were based on different samples and detection methods; this may lead to inaccurate diagnosis of infection, especially considering that questionnaire and clinical diagnosis may be quite unreliable. Fifth, infection of parasites was not analyzed in this study. Recently, infection of *Toxoplasma gondii* was found to increase the risk of PD (Ramezani, Shojaii, Asadollahi, Karimialavijeh, & Gharagozli, 2016), indicating that infection of parasites may also contribute to PD. Finally, none of the included studies stated whether the infection was acute, chronic, or latent.

This meta‐analysis confirmed that infection including HP, HCV, Malassezia and pneumoniae may increase the risk of PD. Antiviral treatment of HCV reduced the risk of PD. Our meta‐analysis suggests that infection of some pathogenic microorganisms may increase the risk of PD, but more studies with high quality are needed before they could be applied in clinic.

## CONFLICT OF INTEREST

All authors declare that they have no conflict of interest.

## Data Availability

The data that supports the findings of this study are available in the tables of this article.
